# Application of Calcium Sulfate for Dead Space Management in Soft Tissue: Characterisation of a Novel In Vivo Response

**DOI:** 10.1155/2018/8065141

**Published:** 2018-03-06

**Authors:** Rema A. Oliver, Vedran Lovric, Chris Christou, Sean S. Aiken, John J. Cooper, William R. Walsh

**Affiliations:** ^1^Surgical & Orthopaedic Research Laboratories, Prince of Wales Clinical School, Prince of Wales Hospital, UNSW Sydney, Randwick, NSW, Australia; ^2^Biocomposites Ltd., Keele Science Park, Keele, UK

## Abstract

Management of dead space (DS) is a fundamental aspect of surgery. Residual DS following surgery can fill with hematoma and provide an environment for bacterial growth, increasing the incidence of postoperative infection. Materials for managing DS include polymethyl-methacrylate (PMMA), which is nonresorbing and requires removal in a second surgical procedure. The use of calcium sulfate (CS) offers the advantage of being fully absorbed and does not require subsequent surgical removal. As CS has historically been used as a bone void filler, there are some concerns for the risk of heterotopic ossification (HO) when implanted adjacent to soft tissue. This study assessed the osteoinductive potential of CS and identified and characterised residual material present in muscle tissue using histology, energy-dispersive X-ray spectroscopy analysis, and scanning electron microscopy (SEM). CS beads with and without antibiotic were implanted in intramuscular sites in both athymic rats and New Zealand white rabbits. At 28 days after implantation in the rat model, no signs of osteoinduction were observed. In the rabbit model, at 21 days after implantation, almost complete bead absorption and presence of a “halo” of material in the surrounding muscle tissue were confirmed. Our results suggested that the halo of material was a calcium phosphate precipitate, not HO.

## 1. Introduction

The importance of effective surgical management of dead space as a result of debridement in procedures to treat periprosthetic joint infection and osteomyelitis cannot be underestimated and is an essential aspect of clinical practice in septic surgery [1]. The use of muscle flaps to minimise soft tissue dead space is effective [2–4], but residual dead space can remain which can fill with hematoma, an ideal environment for bacterial growth. This can increase the chance of infection reoccurrence. Also, the use of local tissue flaps and free flaps may not be possible if there has been extensive debridement.

Antibiotic impregnated PMMA beads have been used as a dead space filler for over 30 years [5] and PMMA is frequently applied as a spacer in joint revision surgery [6, 7], or as beads on a wire or in a pouch to facilitate their removal [8]. PMMA requires surgical removal to prevent it becoming a potential nidus for future infection [9, 10].

The use of CS as a material for management of residual dead space offers important advantages as it is resorbed* in vivo* [11, 12] and does not require subsequent surgical removal. CS can also be combined with antibiotics to treat infections [13]. CS has historically been used as a bone void filler [14, 15]. However, literature has reported that CS, by itself, does not stimulate bone formation. New bone formation occurs when periosteum or bone is also present [16]. Biocompatibility was evaluated by implantation of CS into the proximal epiphysis of the rabbit tibia [17]. The authors found that CS did not induce an inflammatory reaction and was quickly resorbed. It did not support bone formation unless in contact with bone tissue supporting an osteoconductive mechanism. Another animal study concluded that when implanted into subcutaneous sites, CS resorbed too rapidly to be effective in inducing bone replacement [18].

Despite the data available, there remain some concerns regarding the risk of heterotopic ossification (HO) when CS is implanted in soft tissue [19, 20], which could complicate its use in dead space management. Note there are calcium phosphates that have been shown to be inductive in muscle [21].

In our previous studies we developed a novel animal model to investigate dead space management in soft tissue [22]. The study assessed the performance of a recrystallised, pharmaceutical grade CS material and found that it is effective in resolving the surgically created dead space and did not elicit any unexpected adverse host response.

The aim of the current study was to identify and characterise any residual material present in muscle tissue using histology, energy-dispersive X-ray analysis (EDXA), and scanning electron microscopy (SEM) in a rabbit intramuscular model for 21 days.

An osteoinduction assay was performed according to ASTM 2529-13 using CS beads implanted for 28 days intramuscularly in athymic rats to examine for any inductive capabilities.

## 2. Materials and Methods

### 2.1. Preparation of Implant Materials

As previously described [20], under sterile conditions, 10 mL kits containing 20 g of synthetic recrystallised calcium sulfate alpha-hemihydrate powder (Stimulan®, Biocomposites Ltd., UK) were used to prepare the beads for implantation; 20 g of calcium sulfate alpha-hemihydrate was mixed with 6 mL of the provided mixing solution for unloaded beads; for the antibiotic containing beads, 20 g of calcium sulfate alpha-hemihydrate was mixed with 1000 mg vancomycin hydrochloride powder (Hospira UK Limited) and thoroughly blended prior to the addition of 6 mL of tobramycin sulfate solution (40 mg/mL, Hospira UK Limited).

**Figure 1:**
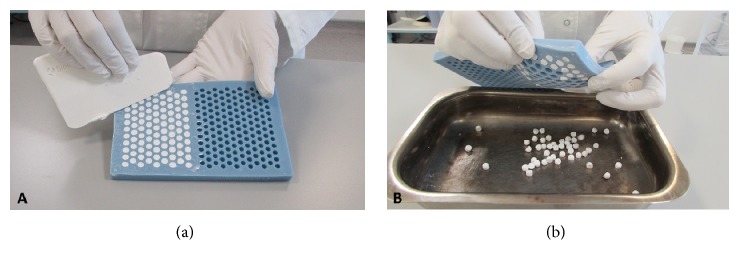
*Preparation of implant material*. (a) Preparation of the calcium sulfate beads using the flexible mould. (b) Removal of beads from mould once set hard [22].

In each case, all components were mixed thoroughly for 30 to 60 seconds to form a smooth paste which was pressed into 6.0 mm diameter hemispherical cavities in the flexible mould with a height of 4.8 mm (Figure 1(a)). The beads were left undisturbed for 10 minutes to hydrate and set according to the following reaction:

(1) $$\begin{array}{c} {CaSO}_{4}\frac{1}{2}H_{2}O+1\frac{1}{2}H_{2}O={CaSO}_{4}2H_{2}O \end{array}$$

When set hard, the beads were removed by flexing the mould (Figure 1(b)).

### 2.2. Surgery

#### 2.2.1. Rabbit Model

An established dead space muscle tissue model was used in two skeletally mature adult New Zealand white rabbits following ethical approval from the Animal Care and Ethics Committee of the University of New South Wales (ACEC #: 13/67A). Under gaseous anaesthesia (isoflurane/oxygen inhalation), two test materials were implanted: beads of CS alone and beads of CS combined with vancomycin and tobramycin (CSVT). The materials (1 cc per site, 5 beads of material) were placed into intramuscular sites following retraction of the longissimus muscles at 4 nonadjacent implant sites.

**Figure 2:**
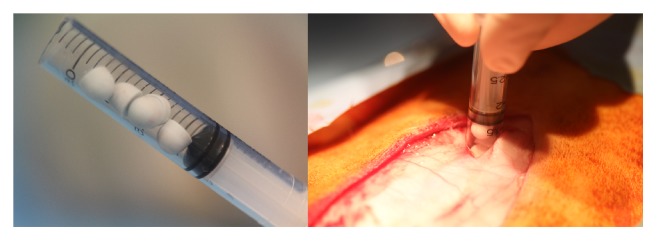
*Surgical implantation of beads*. Five beads preloaded into syringes were implanted into each individual site.

The surgical technique described previously [22] was carried out for both animals in the study. A sample size of *n* = 8 sites was used in this study. Four implant sites were used per animal, two sites on each side of the spine, in nonadjacent intramuscular sites (longissimus muscles) above the levels in L1-L2, L2-L3, L3-L4, and L4-L5 (Table 1). Each site was filled with 1 cc of test material, representing five beads of material per implant site (Figure 2). Each animal received CS beads in two sites and CSVT beads in the remaining two. Both animals were sacrificed at 21 days postoperatively based on our previous study where CS resorption was not complete [22].

The allocation of material to implant sites is outlined in Table 1.

Radiographs were taken with a mobile X-ray machine (Poskom Co., Ltd., Korea) and digital plates (AGFA, Sydney, Australia) to determine resorption.

Twenty-one days after implantation, the animals were euthanised, radiographs and *μ*CT were performed, and the implant sites were inspected and harvested for evaluations to assess appearance, presence of material in the muscle tissue adjacent to the bead implantation, healing, and local tissue reactions. Radiographs were used to identify the harvest sites of any residual material.

MicroCT was performed for all animals using an* in vivo* scanner (Inveon, Siemens Medical, PA, USA) to further examine the intramuscular sites. The surgical sites were scanned and the raw images reconstructed resulting in effective pixel size of 53.12 *μ*m. 3D models were created using Siemens image analysis software (Inveon Research Workplace 3.0, Siemens Medical, PA, USA) to assess* in vivo* absorption of the beads, presence of material in the adjacent muscle tissue, and any adverse tissue reactions. Images were examined in the axial, sagittal, and coronal planes for presence of residual graft material or evidence of new bone formation.

Harvested implant sites were fixed in phosphate buffered formalin for a minimum of 48 hours. Samples were dehydrated in ethanol and embedded in PMMA [23]. Sections were cut using an SP1600 Leica microtome (Leica, Germany) and stained using methylene blue-basic fuchsin to identify any residual material or new bone formation. Stained sections were photographed using an Olympus Microscope (Olympus, Japan) and Olympus DP72 Camera. Identification of the material in muscle tissue was performed on PMMA sections. These were used to examine the material in the muscle tissue using a scanning electron microscopy and energy-dispersive X-ray spectroscopy (EDS). The EDS was used to identify the material present in the intramuscular sites.

#### 2.2.2. Athymic Rat Model

The osteoinduction assay was performed according to ASTM F2529-13 Standard Guide for in vivo Evaluation of Osteoinductive Potential for Materials Containing Demineralized Bone (DBM) [24].

Two nude male rats (age 8 weeks, 240 g) were implanted intramuscularly with CS beads (*n* = 2 sites per rat). The beads were 3 mm diameter hemispherical and were sterilised by gamma radiation. One bead was placed in each site. For each rat, two paravertebral cutaneous incisions were achieved to expose the right and left dorsal paravertebral muscles. At each implantation site, the muscle was gently dissected, taking care to be aligned with the muscle fibre plane to obtain a muscular pocket large enough to insert each 3 mm bead (approximate bead weight = 30 mg). The muscular pocket was sutured using a nonresorbable polypropylene thread (Prolene 4/0, Ethicon) and the skin was closed with stainless steel wound clips. The rats were observed daily for general health. Analgesic treatment (buprenorphine, Buprecare®, Axience) was administered the day after the surgery. Wound clips were removed after complete wound healing two weeks after the surgery. At 28 days after implantation, each rat was euthanised by an intravenous injection of barbiturates (Dolethal®, Vetoquinol). The muscular tissue was macroscopically examined and excised, allowing a sufficient area around the site for histologic preparation. The sites were fixed in 10% v/v neutral buffered formalin (NBF) followed by dehydration in alcohol solutions of increasing concentration, then cleared in xylene, and embedded in polymethyl-methacrylate (PMMA). Two central cross sections (approximately 7 *μ*m thickness) were prepared from each implanted site using the Polycut Leica System (Leica, Germany). One slide was stained with Goldner Trichrome (GT) and the other one was stained with von Kossa. Qualitative and semiquantitative histological evaluation of the osteoinductive performance of each implanted site was performed. Slides were assessed for bone forming elements (chondroblasts, chondrocytes, osteoblasts, osteocytes, cartilage, new bone, and bone marrow) according to the ASTM Standard [24].

## 3. Results

For both models, surgery was completed without any adverse events. All animals recovered well after surgery.

### 3.1. Rabbit Model

During harvest the surgical sites were carefully dissected and examined for the presence of any residual material in the muscle tissue adjacent to the implantation site. No material was visible on macroscopic inspection.

#### 3.1.1. Radiography and *μ*CT

**Figure 3:**
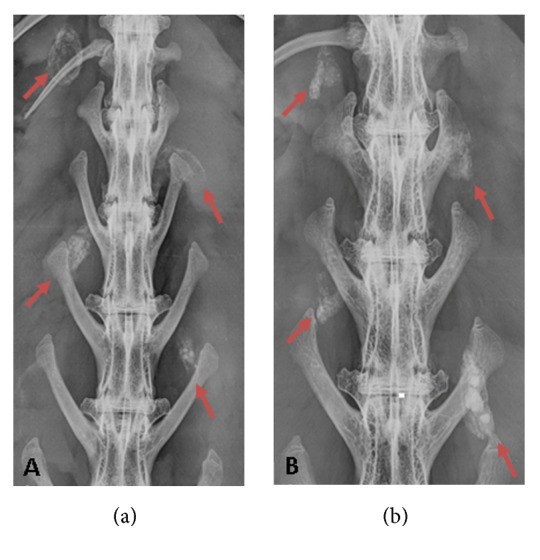
*Radiographic appearance of material*. “Wispy” halo encapsulation was evident at 3 weeks for each implantation site (red arrow) in both animals. Residual calcium sulfate was also present in some of the sites.

**Figure 4:**
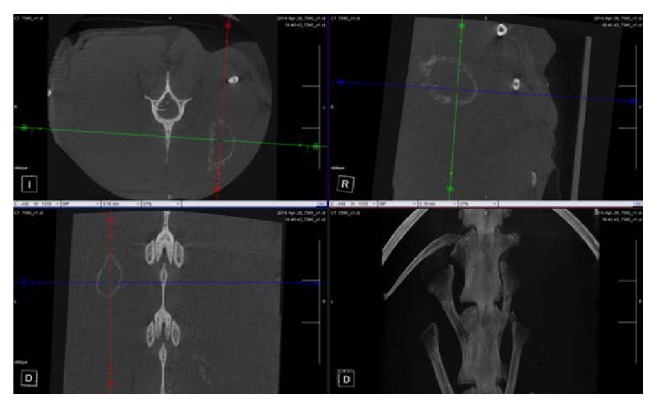
*Micro CT appearance of material*. Confirmation of radiographic findings on *μ*CT demonstrating a “wispy” halo appearance at the implantation sites.

Radiographs and *μ*CT at 21 days showed nearly complete material resorption when compared to postoperative radiographs. The presence of a “wispy” halo of material was evident (Figures 3 and 4) in muscle tissue adjacent to both CS and CSVT beads.

#### 3.1.2. Histology

**Figure 5:**
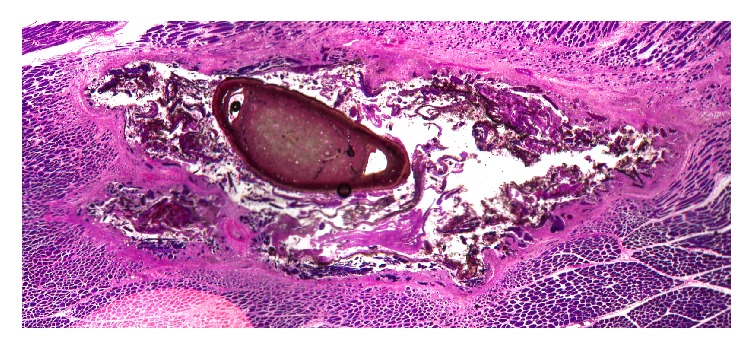
*PMMA embedded undecalcified histology*. Histology revealed the presence of a precipitated residue material surrounded by a fibrous tissue encapsulation. The material did not have the appearance of heterotopic bone. It appeared to be a crystalline deposit, with no noticeable adverse reaction.

The response to both materials was identical in nature and magnitude, all with lymphocytes and red blood cells at the implant/host tissue interface, and multinucleated cells present. The majority of the implanted material had almost completely resorbed in most sites and was replaced by fibrous tissue. The presence of a precipitated residue material was apparent (Figure 5). The material did not have the appearance of heterotopic bone. It appeared to be a crystalline deposit, with no noticeable adverse tissue reactions.

#### 3.1.3. SEM and EDS Analysis

**Figure 6:**
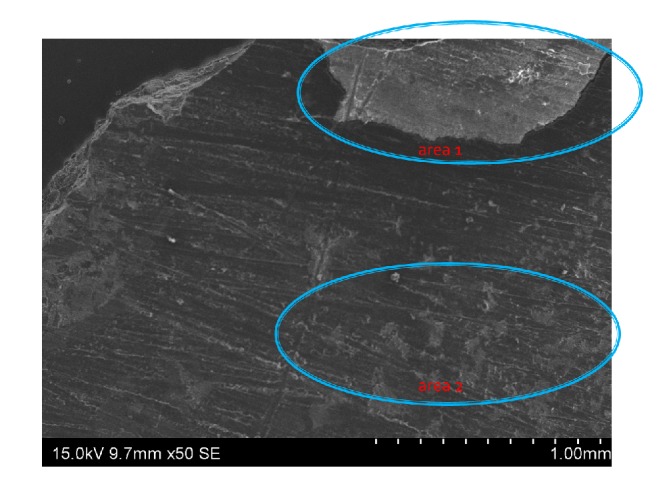
*SEM for two areas of interest*. Area 1 represents implanted calcium sulfate bead while area 2 represents “wispy” white residue material that formed within the soft tissue.

Two areas (area 1 and area 2) were examined with SEM prior to EDS analysis. Area 2 was associated with the residual “wispy” white residue material that had formed in the soft tissue adjacent to the sites implanted with the calcium sulfate beads (area 1) (Figure 6). Analysis confirmed that residue present in the muscle tissue was not calcium sulfate as EDS detected no sulphur peak; therefore no sulfate was present. However, from the identity and ratio of elemental ions present (Ca, P, O) on the scan, the identity of the material was confirmed as predominantly calcium phosphate.

### 3.2. Athymic Rat Model

All animals recovered uneventfully following surgery. No adverse tissue effects were detected macroscopically at any site in the athymic model. Radiographic evaluation at 28 days revealed complete* in vivo* resorption of the CS bead or no other radiopacity present at the implantation sites. Routine histology revealed a mild local inflammatory response while no new bone formation was noted.

## 4. Discussion

An established rabbit soft tissue dead space model [22] was used. CS and CSVT beads resorbed within 21 days; however, a “halo” was found in the muscle tissue.

Our previous study [22] revealed that this “halo” was most pronounced at 21 days after implantation and that, by 42 days after implantation, the “halo” was beginning to disappear. By 63 days it was not visible on X-ray. Therefore, the sacrifice point of 21 days was chosen as the optimal time point to determine the identity of this material.

The material was seen on the methacrylate histology slides at 21 days as a crystalline deposit (Figure 5). This material did not elicit an adverse tissue reaction in the muscle tissue. EDS revealed that crystalline deposit consists predominantly of Ca, P, and O atoms indicating a calcium phosphate material. Analysis indicated that the deposit did not contain any residual calcium sulfate as no sulphur peak was present. It can be postulated that this material is a precipitate formed from the local soluble calcium ions from the absorbing calcium sulfate reacting with serum phosphate ions in the extracellular matrix adjacent to the surgical site. These observations have similarity with those from recent research, which attempted to determine the mechanism by which CS supports bone growth. The implantation of CS in a series of* in vitro* and* in vivo* studies (rabbit femur and tibia defect models) was carried out, determining the effects from a few days after implantation up to sixteen weeks [25].

The CS was observed to resorb* in vitro* and* in vivo*, from the outer surface inward at rates of up to 1 mm per week. The stimulation of new bone formation was observed although bone was not observed to come in direct contact with the CS. The study reported that in most animal models CS dissolved completely, sometimes in 4 weeks, leaving behind concentric rings of mineral deposits in surrounding tissue. Histologically, these deposits stained like bone mineral, frequently with attachment of osteoid and new bone, therefore acting as scaffolds for new bone formation. Analysis of these deposits indicated they were calcium phosphate deposits in the form of a precipitated carbonate apatite, very similar to bone mineral in composition. Analysis ruled out immature bone and residual calcium sulfate [25]. Similarly, concentric circular lamella of bone around calcium sulfate pellets in other animal models has been reported in literature [26–28].

A similar mechanism may be occurring following implantation in soft tissue. However the calcium phosphate precipitate does not result in bone formation supporting the findings from previous studies, where calcium sulfate only stimulated new bone formation where periosteum or bone was also present [16–18]. Therefore, considering the findings from these previous studies, it is suggested that the precipitate is only osteoconductive and not capable of an osteoinductive response. This is supported by the observations that heterotopic ossification was not observed in this study, or when the same model was reported with calcium sulfate implantation in 24 rabbits [22].

The development of HO can be associated with a number of predisposing factors, including major joint surgery, with hip, elbow, and shoulder being the most common joints affected [29]. It is also problematic in amputation in combat personnel [30, 31]. Due to its complex aetiology, a direct link between the placement of calcium sulfate in or adjacent to soft tissue and the occurrence of HO cannot be made. HO has been observed following joint revision surgery [32, 33]. In a consecutive series of 135 patients undergoing revision total knee arthroplasties, the only risk factor identified for the development of HO was the presence of infection (76%). This was significantly higher than the 47% incidence of HO in patients who did not have an infection. The study also found no associated risk of HO with factors such as patient size (body mass index), surgical time, operative approach, or number of prior knee procedures [32].

The possibility of the presence of a precipitated calcium phosphate in muscle tissue increasing the risk of HO when other factors are present cannot be completely ruled out and warrants further study.

The limitations of this study are that only the materials were implanted in soft tissue sites and do not directly compare with the* in vivo* response in a bone tissue implantation site in rabbits. The potential effect of the antibiotics combined with CS on the osteoconductivity was therefore also not assessed. A small number of animals were used (2 rabbits and 2 rats) and only one commercially available calcium sulfate material was used. Lastly, the soft tissue sites were not debrided, which is common in clinical management of dead space to remove necrotic tissue. The presence of local bleeding and effects of soft tissue debridement were therefore not assessed.

## 5. Conclusions

In the osteoinduction assay, CS was not shown to be osteoinductive, an important consideration when these materials are implanted in soft tissue as part of a dead space management strategy. The transient material which was seen to form in the rabbit soft tissue model following absorption of the CS beads was identified as a precipitated calcium phosphate and not due to the formation of HO.

The* in vivo* response to CS is more complex than a simple dissolution of CS in the soft tissues. The occurrence and the transient nature of this precipitate, as supported by a previous study of CS in this model [22], may also prove informative to clinicians when reviewing plain radiographs of patients who have been implanted with CS in soft tissue.

The material behaviour in these animal models should not be taken as indicative of clinical performance in human subjects. As mentioned in the limitations, the soft tissue sites were not debrided, which is common in clinical management of dead space to remove necrotic tissue. The presence of local bleeding and effects of soft tissue debridement were therefore not assessed.

Future studies to consider will include comparative implantation of these materials in bone and soft tissue sites, as well as the effect of different antibiotics and antibiotic dosing, in combination with CS on the* in vivo* response.

## Figures and Tables

**Table 1:** Study design and implantation schematic [22].

Animal	Surgical site	Implant material
(1)	L1-L2 left side	CSVT
	L2-L3 right side	CS
	L3-L4 left side	CSVT
	L4-L5 right side	CS

(2)	L1-L2 left side	CS
	L2-L3 right side	CSVT
	L3-L4 left side	CS
	L4-L5 right side	CSVT

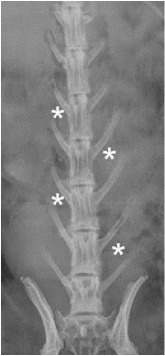		

One time point with 2 animals with 4 implantation sites per animal (asterisks).
